# Strong Emission Enhancement via Dual-Wavelength Coexcitation in YbTm-Doped Upconverting Nanoparticles for Near-Infrared and Subdiffraction Imaging

**DOI:** 10.1021/acsnano.5c08510

**Published:** 2025-07-08

**Authors:** Paulina Rajchel-Mieldzioć, Artur Bednarkiewicz, Katarzyna Prorok, Piotr Fita

**Affiliations:** † Institute of Experimental Physics, Faculty of Physics, 49605 University of Warsaw, Pasteura 5, Warsaw 02-093, Poland; ‡ 215275 Institute of Low Temperature and Structure Research, Polish Academy of Sciences, Okólna 2, Wrocław 50-422, Poland

**Keywords:** luminescent materials, upconversion, nanoparticles, nanophotonics, near-infrared detection

## Abstract

Upconversion (UC) emission in lanthanide-doped nanoparticles is typically excited by a single near-infrared (NIR) wavelength, most commonly around 975 nm, which promotes ground-state absorption by Yb^3+^ sensitizer ions and subsequent energy transfer to activator ions such as Tm^3+^. However, due to the presence of multiple long-lived excited states in lanthanide ions, additional excitation wavelengths can activate or modulate further energy-transfer pathways, leading to enrichment or depletion of specific electronic level populations. Despite their significant potential, such possibilities remain underexplored. In this work, we present a dual-wavelength coexcitation approach applied to NaYF_4_ upconverting nanoparticles (UCNPs) codoped with Yb^3+^ and Tm^3+^ ions, using simultaneous illumination with a primary 975 nm beam and a secondary, tunable NIR beam. We observed that the addition of NIR light at wavelengths corresponding to two absorption bands of Tm^3+^, centered at 1213 and 1732 nm, to conventional 975 nm excitation yields a strong emission enhancement (up to 800%—far exceeding the additive effect of each excitation wavelength alone). Strikingly, in certain instances, *de novo* emission is observed under coexcitation, even when neither excitation source alone produces detectable luminescence. We show that this coexcitation mechanism enables visualization of NIR light above 1700 nm, beyond the detection range of silicon and standard InGaAs photodetectors. Furthermore, it allows the modulation of UC emission at excitation intensity levels that are several orders of magnitude lower than those used in super-resolution techniques such as STED. These findings indicate that coexcitation can become a powerful and energy-efficient method for controlling UC emission, with direct implications for subdiffraction imaging, NIR sensing, and advanced photonic applications.

## Graphical abstract

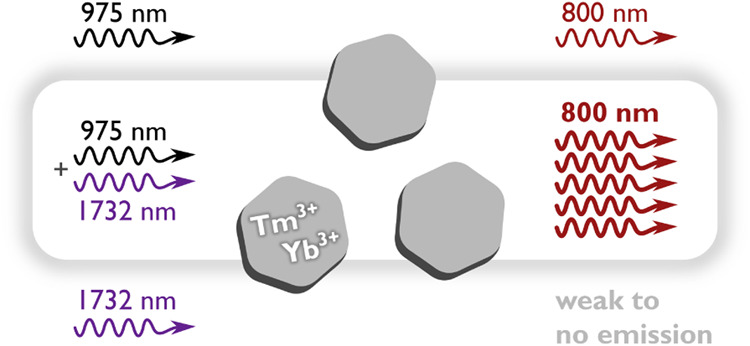

## Introduction

Upconverting nanoparticles (UCNPs) are a class of inorganic luminescent materials that absorb lower-energy photons—typically in the near-infrared (NIR) region—and emit higher-energy photons in the visible or ultraviolet range.^1^ The upconversion phenomenon is enabled by the presence of multiple closely spaced manifolds in the 4*f* shells of rare-earth ions. Due to the parity-forbidden nature of *f*–*f* transitions, these ions exhibit long-lived intermediate excited states, allowing sequential photon absorption if the photon energies are appropriately matched to transitions along the ladder of excited states. The upconversion has been observed in single dopants, but employment of sensitizer ions, typically Yb^3+^, enhanced the efficiency by 10–100-fold in the process of energy-transfer upconversion (ETU).^2^ It relies on the sequential absorption of photons through sensitizer–activator interactions.^2–4^ The Yb^3+^ sensitizer is particularly effective due to its large absorption cross section at around 975–980 nm, low susceptibility to concentration quenching, and efficient energy-transfer properties owing to a simple energy structure and long-lived metastable level. In a typical ETU process, Yb^3+^ ions are excited via the ^2^F_7/2_→^2^F_5/2_ transition (Figure 1) by 980 nm photons and subsequently transfer the absorbed energy to nearby activator ions, promoting them to their metastable excited states and resulting in upconverted emission. As a result, rare-earth ion codopant pairs such as Yb^3+^, Er^3+^, and Yb^3+^, Tm^3+^, embedded in a crystalline host lattice, successively populate their 4*f* excited states of the activators before emitting a photon of higher energy than the single excitation photon.

**Figure 1:**
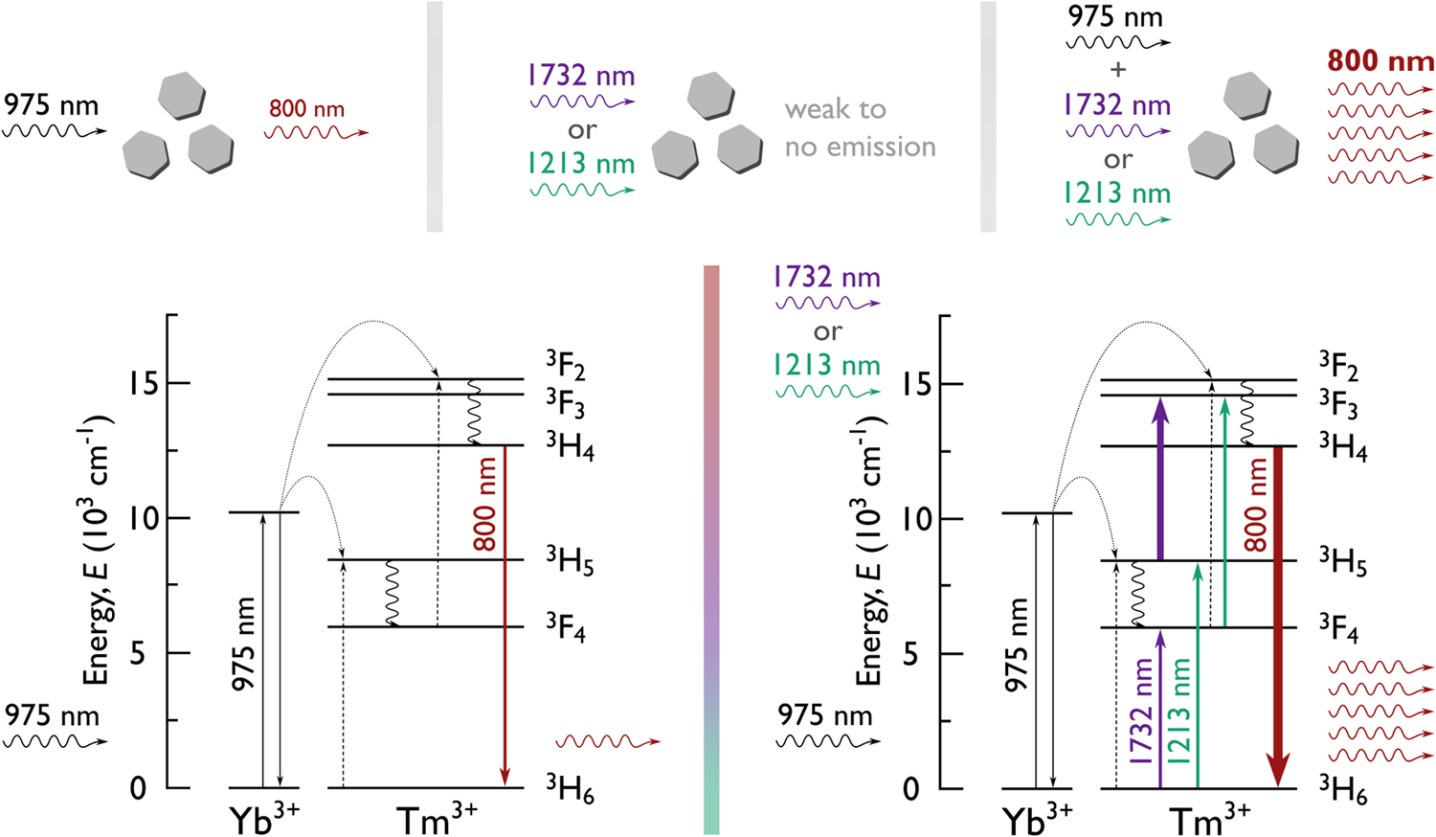
Illustration of the concept behind coexcitation of thulium-doped nanoparticles using a primary 975 nm beam and an additional NIR beam. **Top:** a schematic representation of how specific laser excitations influence the 800 nm emission from the nanoparticles. Excitation with the 975 nm beam alone induces emission; excitation with any of the selected NIR beams alone (corresponding to absorption bands identified and described in this work) results in negligible or no measurable emission. However, coexcitation with the 975 nm beam and one of the selected NIR wavelengths leads to a significantly enhanced 800 nm emission. **Bottom:** energy-level diagrams illustrating the observed transitions. The thickness of the absorption transition lines in thulium ions, associated with transitions made accessible by the introduction of a NIR beam, approximately reflects the oscillator strength as described by the Judd–Ofelt theory.^15,16^

In addition to wavelengths typically used for UCNP excitation, obviously there are others that match specific transitions in emitting ions alone. Therefore, one can expect that simultaneously exciting UCNPs at multiple wavelengths—especially those precisely tuned to the relevant electronic transitions—will open additional photoexcitation channels and influence the emitted light intensity, potentially leading to previously unexplored phenomena and applications.^5^ In such coexcitation schemes, the emission intensity should be a nonlinear function of the intensities of all exciting beams. This nonlinearity could be utilized for applications in optical microscopy, where resolution and contrast may be improved in scattering media as well as in all-optical and neuromorphic computing, nanolithography, temperature, force sensing, or biosensing.

The rapid development of near-infrared, compact diode lasers has made wavelengths in the 1000–1700 nm range readily available, often with fine steps of 5–20 nm. Two or more such lasers can be easily combined for coexcitation of UCNPs, making this approach not only potentially useful but also experimentally feasible and cost-effective. Despite these advantages, studies on the photophysics of UCNP coexcitation remain sparse,^6,7^ and existing applications have largely focused on stimulated emission depletion (STED) super-resolution microscopy.^5,8–11^

In this work, we investigated coexcitation in well-established UCNPs sensitized by ytterbium ions (Yb^3+^) and using thulium (Tm^3+^) ions as activators. YbTm UCNPs have already been used in STED microscopy, where coexcitation at 975 and 810 nm resulted in a remarkable depletion exceeding 90% at relatively large pump (0.66 MWcm^–2^) and depletion (>10 MWcm^–2^) beam intensities, respectively.^9^ In the present study, we focused on the near-infrared range by coexciting 20% Yb^3+^- and 0.1% Tm^3+^-codoped UCNPs with a continuous-wave laser at 975 nm and a tunable beam ranging from 1050 to 1875 nm. We identified two distinct bands (centered at approximately 1213 and 1732 nm; Figure 1) that enable the efficient coexcitation of YbTm nanoparticles and demonstrated that this near-infrared coexcitation leads to pronounced enhancement in upconversion emission—or even enables signal generation under conditions where neither excitation source alone produces a detectable response, thus allowing for visualization and detection of NIR radiation beyond the spectral sensitivity range of standard InGaAs detectors (above 1700 nm). Additionally, it allows for the precise control of the UCNP response to one stimulus (excitation at one wavelength) through the presence and intensity of the second stimulus (excitation at another wavelength).

The effects described in this work can become the basis for numerous photonic applications, such as all-optical computing,^12^ exemplified by the straightforward realization of an AND logic gate (emission is observed under coexcitation) or transistor-like optical performance (controlling pristine luminescence with *gate* coillumination). Furthermore, our results indicate that the coexcitation of YbTm nanoparticles may facilitate novel variants of super-resolution microscopy using only NIR beams, conceptually similar but distinct from conventional STED^13,14^ approaches.

## Results and Discussion

When excited by a 975 nm beam, the analyzed YbTm nanoparticles exhibit a characteristic visible spectrum (Figure S1a,b), featuring a prominent maximum at the relatively low-energy emission of 800 nm. This emission wavelength provides a reference for assessing the nonstandard excitation pathways along the 4*f* manifolds of Tm^3+^. Figure 2a displays the coexcitation spectrum (recorded at an emission wavelength of 800 nm) of YbTm nanoparticles excited by a continuous-wave 975 nm beam (10 mW; 0.3 kW/cm^2^) in combination with a tunable NIR beam (100 mW; from 3.8 kW/cm^2^ at 1213 nm to 3.0 kW/cm^2^ at 1732 nm), plotted as a function of the wavelength of the latter. For reference, the same plot shows the baseline emission intensity induced by the 975 nm beam alone along with the emission intensity generated by the NIR beam alone.

**Figure 2:**
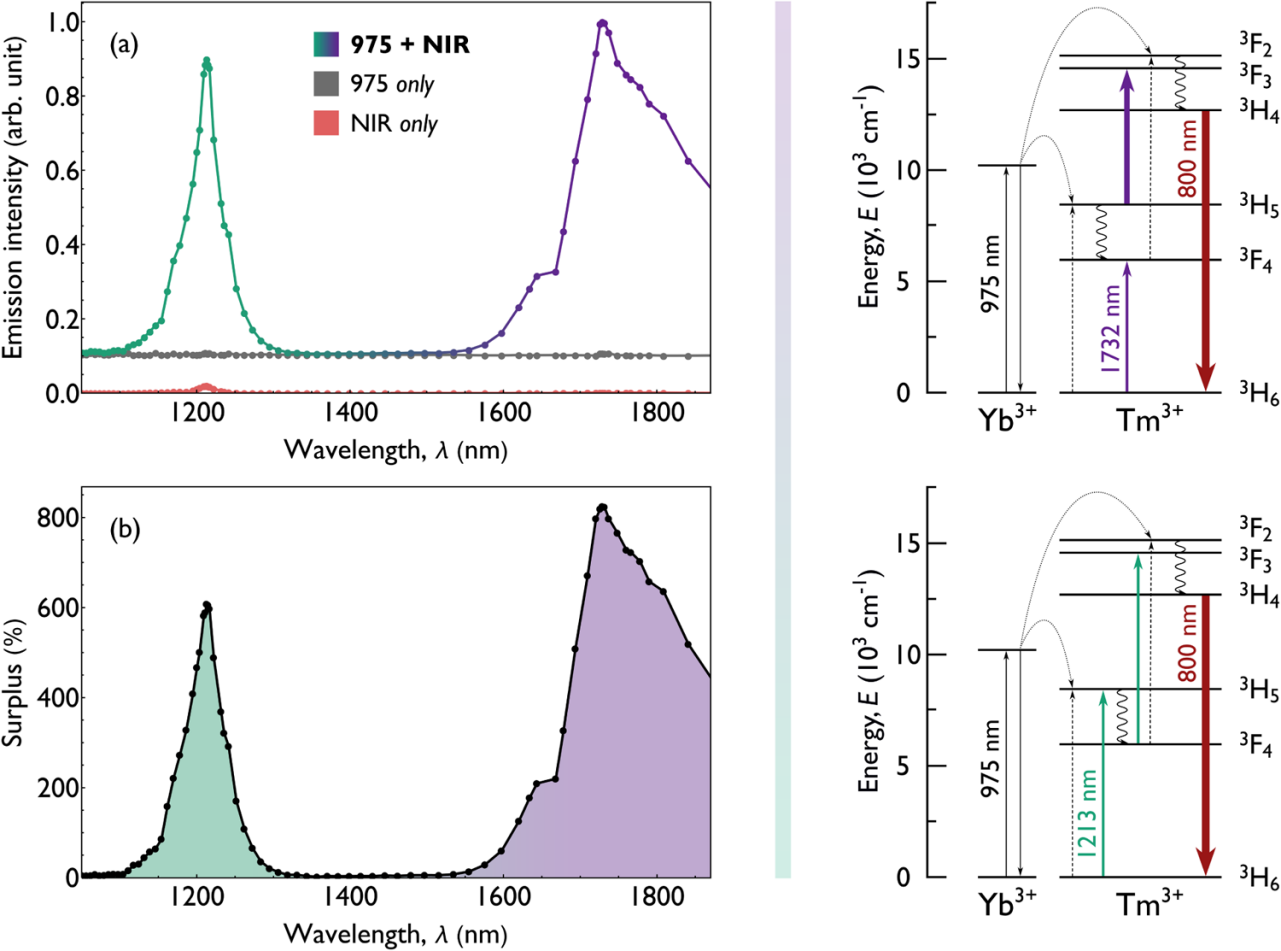
**Left:** excitation spectra of YbTm nanoparticles under simultaneous excitation with two collinear beams: a fundamental 975 nm beam (10 mW; 0.3 kW/cm^2^) and a complementary NIR beam (100 mW; from 3.8 kW/cm^2^ at 1213 nm to 3.0 kW/cm^2^ at 1732 nm). (a) Excitation spectra recorded at an 800 nm emission, measured under three excitation conditions (all shown at the same scale, normalized to the maximum measured value): (i) coexcitation with both beams (bichromatic green-violet line, to better distinguish the two distinct bands, corresponding to analogously color-coded transitions), (ii) excitation with only the NIR beam (coral line, positioned near the bottom, with nonzero values only around 1200 nm), and (iii) the baseline 800 nm emission induced by the 975 nm beam alone (gray, the points correspond to the current setting of the NIR beam, even though it was blocked during this measurement). The emission intensity is plotted as a function of the NIR beam wavelength. (b) Surplus emission plot under coexcitation, showing the enhancement relative to the **sum** of 800 nm emission intensities generated by each beam alone. **Right:** simplified energy diagrams of the YbTm system, illustrating the ground-state absorption (GSA) and possible excited-state absorption (ESA) processes that govern the observed excitation spectra.

The coexcitation spectrum reveals two distinct bands, with maxima at 1213 and 1732 nm. A review of the thulium energy-level diagram (Figure 2) suggests several viable wavelength options that can effectively interact with the selected nanoparticles while simultaneously being populated via energy-transfer upconversion (ETU) from ytterbium sensitizer. When the Judd–Ofelt theory and the theoretically calculated reduced matrix elements are considered for given Tm^3+^ transitions, there are many ground-state absorption (GSA) and excited-state absorption (ESA) transitions of considerable probability. For example, the GSA ^3^H_6_ → ^3^H_5_ and ESA ^3^F_4_ → ^3^F_3_ transitions should exhibit comparable absorption cross sections. Similarly, GSA ^3^H_6_ → ^3^F_4_ and ESA ^3^H_5_ → ^3^F_3_ should be similarly efficient. However, in steady-state and room-temperature conditions, only ground states are populated, and only GSA transitions are observed, while ESA transitions critically depend on the, typically negligible, population of the excited states (^3^F_4_ and ^3^H_5_). The ESA cross section varies with the host matrix, local crystal field, and temperature, but on top of that, it will strongly depend on additional population through 975 nm sensitized Yb^3+^ ions. This explains why these wavelengths (1213 and 1732 nm) alone do not independently produce significant visible emission—particularly in the case of the 1732 nm band (Figure S2). Coexcitation is further supported by the forbidden nature of the excited states, resulting in their long lifetime, which allows the energy transferred from the Yb^3+^ sensitizer to be temporarily stored until NIR photons arrive and promote the electrons to higher-energy levels via the excited-state absorption (ESA) process. A very weak emission is observed with excitation around 1213 nm, which can be attributed to the relatively higher likelihood of 1213 nm photons exciting the upper 4*f* manifolds of thulium. This occurs via an ESA transition from ^3^F_4_ to ^3^F_3_, where the ^3^F_4_ state is initially populated through the GSA followed by relaxation. In contrast, excitation at 1732 nm primarily populates the ^3^F_4_ state via GSA, but it does not sufficiently populate the ^3^H_5_ state, which is critical for enabling the subsequent ESA transition ^3^H_5_ → ^3^F_3_.

From a fundamental perspective, the magnitude of the observed effect exceeded expectations, yielding a much stronger enhancement than initially projected; the spectra shown in Figure 2a reveal a strikingly large increase of the emission intensity when both beams are combined, compared to the effect produced by each individual beam separately. The enhancement encompassed all detectable emission bands (Figure S1c,e), but the effect was most pronounced for the low-energy 800 nm emission. Combined with its initial dominance over the other bands, this resulted in the highest final intensities—both absolute and relative—achieved under coexcitation. Accordingly, the analysis focused primarily on this emission wavelength. To fully convey the scale of this effect, Figure 2b shows a plot of the surplus 800 nm emission as a function of the NIR beam wavelength. Here, the surplus *S*_λ_ refers to the percentage increase in emission due to collinear coexcitation, relative to the sum of emission intensities originating from the excitation by each of the beams alone

1 $$S_{\lambda }=\frac{E_{\mathrm{co}-\mathrm{ex}}\left(\lambda \right)-\left(E_{\mathrm{NIR}}\left(\lambda \right)+E_{975}\right)}{E_{\mathrm{NIR}}\left(\lambda \right)+E_{975}}\times 100\%$$

where *E*_co–ex_ is the emission intensity at 800 nm recorded with coexcitation, *E*_NIR_ is the emission intensity for excitation with the NIR beam only, and *E*_975_ is the emission intensity for excitation with the 975 nm beam only; λ (nm) is the current wavelength of the NIR beam.

For the identified spectral bands at the given beam intensity levels, this surplus reaches several hundred percent compared to the baseline emission induced by the 975 nm beam alone. The observations described above motivate another attempt to quantitatively characterize the effect of the surplus emission, which arises from the nontrivial interplay between the intensities of both excitation beams. Figure 3a,b illustrates the dependence of a surplus 800 nm emission on the intensity of the NIR beam (1732 and 1213 nm) for various intensity levels of the 975 nm beam (alternative visualizations of this dependence, shown on both linear and logarithmic scales, are presented in Figures S3 and S4). In particular, increasing the intensity of the 975 nm beam leads to saturation, reducing the influence of the NIR beam on the population dynamics of the Tm^3+^ 4*f* manifolds. While surplus emission increases with the NIR beam intensity, it also exhibits self-limiting behavior, becoming less pronounced at higher intensities.

**Figure 3:**
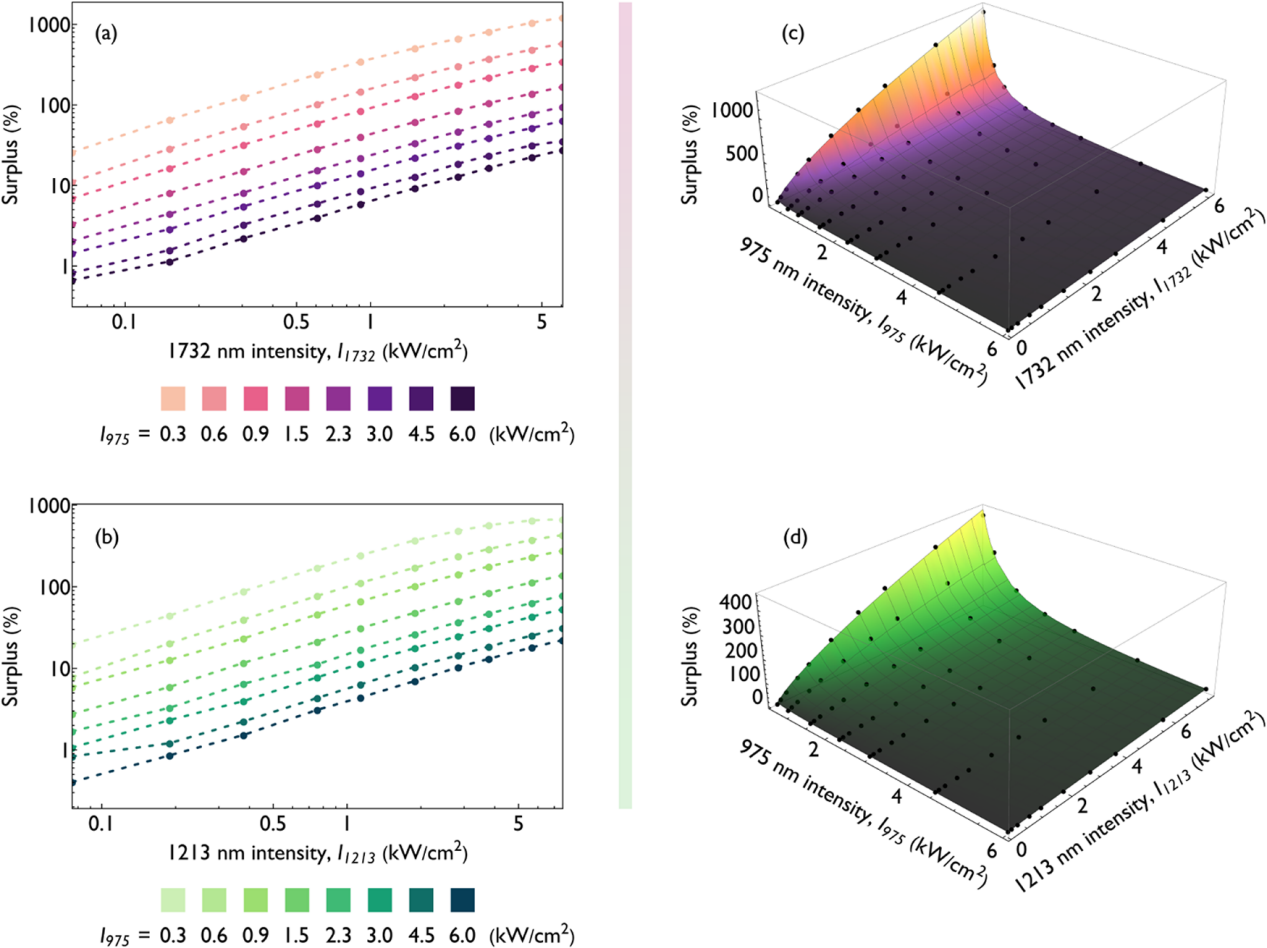
Influence of 975 nm and NIR beam intensity on the 800 nm emission intensity of YbTm nanoparticles under coexcitation. (a, b) Surplus emission from nanoparticles excited simultaneously by the 975 nm beam and (a) 1732 or (b) 1213 nm beam, plotted as a function of the NIR beam intensity for selected intensity levels of the 975 nm beam (dashed lines are an aid for the eye). (c, d) Surplus emission from nanoparticles excited simultaneously with a 975 nm beam and a (c) 1732 or (d) 1213 nm beam (black dots), plotted as a function of the intensity of both excitation beams. The fitted function is represented by the two-dimensional power-law surface.

Although these trends are qualitatively evident from the preliminary analysis, we were able to further quantify the effect by fitting a two-variable power-law function that relates surplus emission to the intensity of both excitation beams, *P*_975_, and *P*_NIR_. Importantly, this function does not include a constant term, ensuring that the dependence is purely multiplicative (eq 2)­

2 $$S_{\lambda }=a\cdot I_{\mathrm{NIR}}^{x}\cdot I_{975}^{y}\Rightarrow S_{\lambda }\;\propto I_{\mathrm{NIR}}^{x}\cdot I_{975}^{y}$$

The surplus is expressed as a percentage, while individual intensity levels are given in kW/cm^2^. The fitted parameters (along with their uncertainties) for the experimental results corresponding to the 1732 and 1213 nm beams are

3 $$S_{1732}=86\left(2\right)\cdot I_{1732}^{0.69\left(1\right)}\cdot I_{975}^{-1.20\left(1\right)}$$

4 $$S_{1213}=54\left(1\right)\cdot I_{1213}^{0.73\left(1\right)}\cdot I_{975}^{-1.30\left(2\right)}$$

As anticipated from preliminary observations, increasing the intensity of the 975 nm beam reduces the observed effect. Although this may seem counterintuitive, it is important to recall that surplus emission is conceptually distinct from total emission intensity, as it represents the deviation of the total measured emission from the sum of the emissions expected if each beam acted independently. In contrast, the absolute emission intensity retained a superlinear dependence within the examined intensity range.

Considering the conclusions drawn directly from the presented analysis, it becomes clear that the surprising magnitude of the effect is not fully captured when using moderate intensities for both beams—especially the primary 975 nm beam. According to eq 2, reducing its intensity leads to a significant increase in the emission surplus. To further demonstrate this, an additional step was taken: both the 975 nm and selected 1732 nm NIR beams were used at intensity levels low enough to produce emission only at the level of dark counts.

While this approach prevents the quantitative calculation of any surplus (since, in the absence of a signal from either beam alone, the surplus tends toward infinity), it clearly demonstrates the potential for direct conversion of the NIR beam to the visible range (indirect NIR detection)—without any detectable signal prior to combining the beams. The experiment not only confirmed that such an effect occurs in practice but also revealed its unexpectedly large magnitude.

Figure 4a,b presents two experiments in which the intensity level of the 975 nm beam alone did not produce any measurable emission (counts remained at the level of dark noise). When combined with the 1732 nm beam—whose contribution at the given intensity levels also remains within the dark count range, a clear and measurable emission is observed. This increase cannot be described in terms of the surplus defined by eq 1 due to the absence of any standalone signal from the individual beams—the denominator of the expression in eq 1 equals 0.

**Figure 4:**
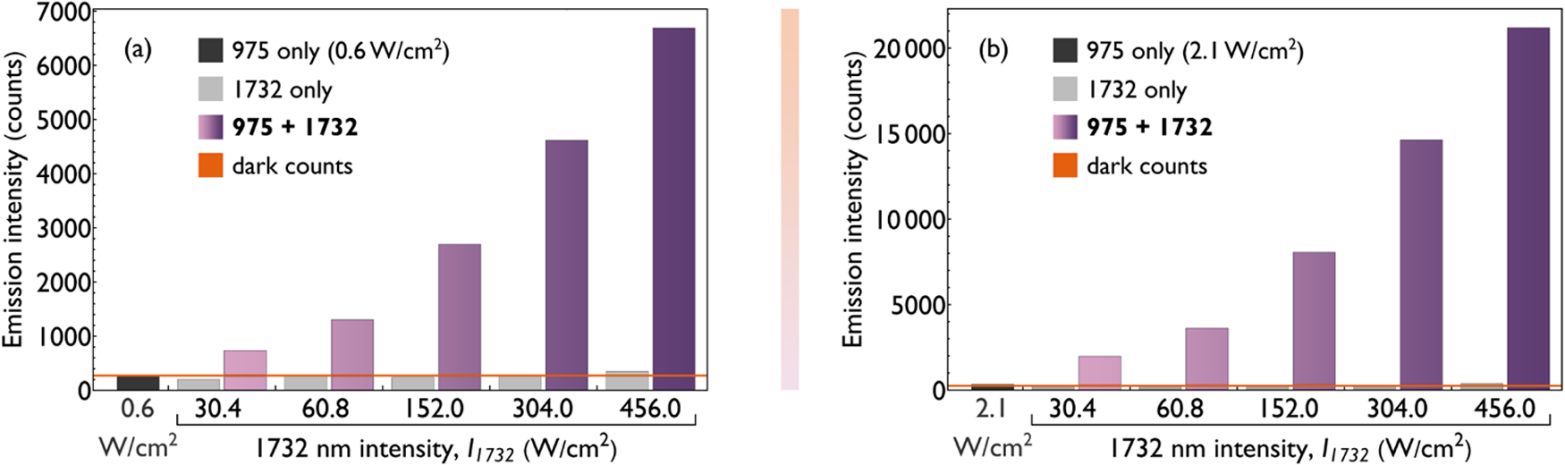
Analysis of an 800 nm emission intensity recorded under coexcitation with weak 975 and 1732 nm beams (purple bars), collected for a range of 1732 nm beam intensities. To illustrate the scale of the effect, the emission intensity under excitation with only the 975 nm beam (dark gray) and only the 1732 nm beam (light gray) is also shown. Emission without beam combination remained at or near the dark count level (orange line). (a) Experiment performed with a 975 nm beam intensity of 0.6 W/cm^2^ (20 μW), while varying the intensity of the 1732 nm beam. (b) Experiment performed with a 975 nm beam intensity of 2.1 W/cm^2^ (70 μW), while varying the intensity of the 1732 nm beam.

To fully appreciate the scale of the effect, the data shown in Figure 4a,b, along with additional measurements performed at a slightly higher 975 nm intensity, are plotted on a logarithmic scale in Figure S5. These results demonstrate that by carefully tuning the intensity of both excitation beams, the observed emission intensity enhancement can be extended up to several orders of magnitude, even enabling entirely *de novo* emission, triggered only by the combined presence of the two individually subthreshold beams.

Our comprehensive analysis, beginning from high-intensity regimes down to the lowest detectable coexcitation levels, clearly illustrates the full dynamic range of the effect. This lays the groundwork for practical applications, enabling control over emission from intense luminescence down to fully switchable states governed solely by beam combination. Regardless of the specific application directions explored in the following sections, it is worth emphasizing that we have demonstrated how strong emission can be generated exclusively through beam combination—where neither of the two beams alone produces any signal detectable by the detector used.

A potential application of YbTm nanoparticles emerges here; excitation with a 975 nm light makes visible emission of these UCNPs sensitive to the presence and intensity of NIR radiation with wavelengths starting at 1200 nm and exceeding 1700 nm. We demonstrated how this observation can be used for the detection and visualization of NIR radiation at wavelengths that are outside the sensitivity range of standard InGaAs semiconducting detectors. As a proof of principle, we prepared a phosphor screen by depositing a thin layer of YbTm UCNPs and illuminated it with a defocused 975 nm light and a NIR beam with its central wavelength set to 1732 nm. Under appropriate conditions (i.e., fixed pump intensities along the whole experiments and switching respective beams on and off only), we observed that neither the fundamental 975 nm beam nor the 1732 nm beam alone produced any visible emission of nanoparticles—whether to the naked eye or captured by a camera. However, when combined, these beams generated a strong red emission, precisely revealing the position of the NIR beam and allowing the determination of its spatial profile with high fidelity (Figures 5a,b, S6, and S7).

**Figure 5:**
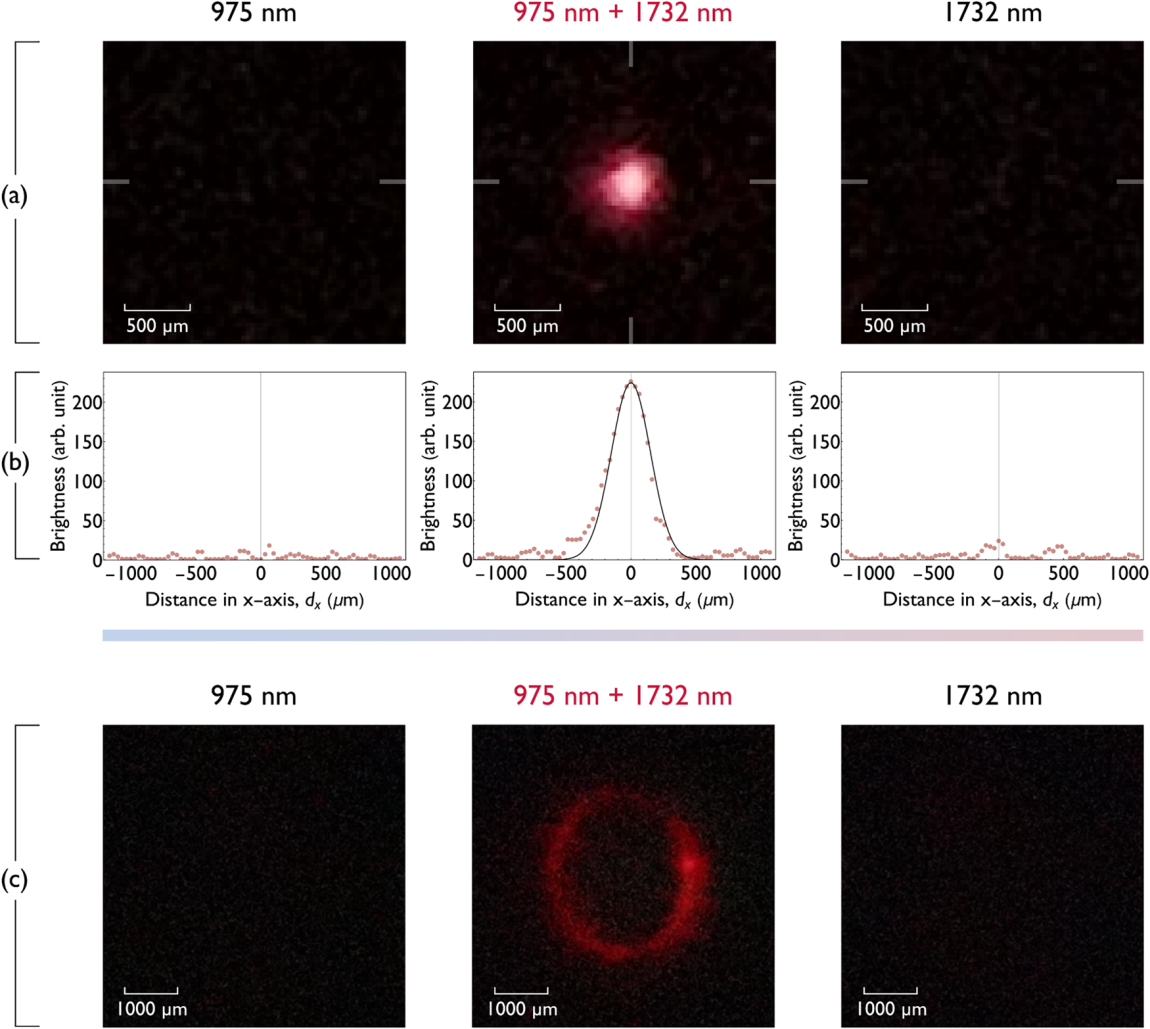
Direct visualization of NIR (1732 nm) radiation using YbTm UCNPs using coexcitation. (a) Photographic image of a glass slide covered with UCNPs excited with a defocused 975 nm beam **(left)**, coexcited with the NIR beam and the defocused 975 beam **(center)**, and with the NIR beam only **(right)**. (b) Horizontal cross sections through images shown in panel (a). On the vertical axis of the graphs is the brightness of each pixel in the original image. The recorded beam profile is fitted with the Gaussian function with fwhm = **355**(13) μm. The signal-to-noise ratio (SNR) of the image was estimated as 64 and 71 for horizontal and vertical cross sections, respectively (details and the vertical cross section are presented in the SI). (c) Visualization of an oval plotted on the screen by scanning the NIR beam with a galvoscanner. Each frame is an average of three consecutively acquired photographs. Images were captured with a Sony A7 III camera through long-pass and short-pass filters transmitting only an 800 nm emission of UCNPs.

Following a successful proof-of-concept experiment demonstrating the emergence of visible emission from otherwise nonemissive excitation conditions, a follow-up experiment was conducted. The NIR beam tuned to 1732 nm was directed using a two-dimensional computer-controlled galvanometer scanner. This setup enabled rapid changes in the beam direction along a programmed path, allowing the projection of a two-dimensional image onto a UCNP-covered phosphor screen in a way analogous to that used in commercial laser projectors. Visualization of an oval-shaped plot produced in this manner is shown in Figure 5c (the local variation in intensity is due to inhomogeneity of the phosphor screen, which requires further optimization). This experiment provided compelling evidence that direct beam visualization is also achievable for two-dimensional images. In this approach, a large area of the UCNP-based phosphor screen is uniformly illuminated with a 975 nm beam at an intensity that does not itself induce detectable emission. When an image is projected onto the screen using the NIR beam, it becomes visible and can be recorded using standard silicon-based sensors that are sensitive to 800 nm.

This strong *switchable* upconversion effect shows considerable potential for infrared beam detection, security, and optical communications, particularly in cases where IR signals need to be visualized or monitored. In security settings, they can enhance the detection of covert signals that are otherwise invisible to standard detectors. In optical communications, where the transmission region beyond 1.6 μm is being explored for future systems, the ability to shift these IR wavelengths into more easily detectable spectral ranges enables straightforward and more cost-effective signal integrity diagnostics.

In the simplest application, YbTm-based nanomaterials can enable real-time visualization of laser beams at wavelengths exceeding 1700 nm, where only visualization cards based on slowly reacting thermochromic liquid crystals or expensive detectors based on extended InGaAs are available. Whereas this emission can be seen with the naked eye or photographed with a standard photographic camera for the NIR beam intensity on the order of 1 kW/cm^2^, a much higher sensitivity would be obtained with photon-counting detectors, such as silicon-based avalanche photodiodes, which exhibit maximum sensitivity close to the Tm^3+^ emission at approximately 800 nm.

Moreover, it has been demonstrated that a photodetector with a direct light-to-current conversion can be built using UCNPs and very high sensitivity of such a detector can be achieved by combining microlens arrays (to concentrate photon flux) and plasmonic effects (to augment emission rates).^17^ If such a photodetector is built using YbTm UCNPs, it would be sensitive at wavelengths exceeding 1700 nm by using coexcitation at 975 nm.^18^ Owing to large (anti)­Stokes shifts, combining multiple wavelengths, and detecting 800 nm photons is technically feasible using dichroic mirrors. Its potential applications are further extended by the fact that the NIR sensitivity can be smoothly controlled by varying the coexcitation intensity and even gated by switching the 975 nm irradiation on or off. These capabilities emphasize the broader utility of the switchable YbTm upconversion system presented when coexcitation is employed. Possible applications include NIR sensing, lock-in ultrasensitive detection (by modulating the 975 nm beam), or optical signal amplification at a relatively low complexity. Currently used NIR detectors with sensitivity beyond 1700 nm are based on extended InGaAs material, which requires advanced and costly fabrication procedures due to a lattice mismatch between the sensor and the substrate as well as are often equipped with thermoelectric cooling to reduce the background noise level.^19^

Stemming from the observed surplus effects, i.e., the fact that surplus emission intensity depends on the product of both beams’ intensities, we predict and evaluate another attractive application. The power of the NIR beam has a positive, albeit sublinear, effect on the surplus emission, whereas the increase of the 975 nm beam intensity reduces the surplus emission. This could potentially have important consequences for microscopy, as one can develop a subdiffraction resolution optical microscopy technique with coexcited UC nanoparticles as biofunctionalized luminescent labels. Similarly to the well-known concept of STED microscopy, the NIR beam should have a Gaussian profile, while the focal spot of the 975 nm beam should have a doughnut shape (such as a Laguerre–Gaussian profile). Both beams should be periodically blocked, and the image should be reconstructed from the surplus emission. In principle, the radial intensity profile of the surplus emission *S*(*r*) should follow eq 2, with *I*_NIR_(*r*) and *I*_975_(*r*) representing radial profiles of respective beams

5 $$S\left(r\right)=a_{\lambda }\cdot I_{\mathrm{NIR}}{\left(r\right)}^{x}\cdot I_{975}{\left(r\right)}^{y}$$

Eq 5 should only be considered as an approximation since it diverges by tending to infinity when *r* approaches zero. However, a qualitative representation of the radial profile of surplus emission can still be obtained by assuming that the intensity at the center of the 975 nm beam is not exactly zero but instead takes on a small finite value (Figure S8). The resulting radial profile of surplus emission is clearly narrower than the profiles of both excitation beams, confirming its suitability to achieve subdiffraction resolution imaging.

In principle, the determination of an image would require three acquisitions: one with simultaneous excitation of the sample by both beams and one with each of the two beams individually. However, if the intensity of the NIR beam is kept sufficiently low, then the emission induced by this beam becomes undetectable. In such a case, the number of required acquisitions can be reduced to two: one with both beams and one with the 975 nm beam only.

Although determining the image from 2 consecutive measurements introduces additional technical complexity compared to conventional STED microscopy, the proposed scheme holds promise for subdiffraction imaging at very low laser intensities. It has already been demonstrated that STED microscopy with UCNPs can achieve super-resolution at depletion beam intensities that are over 2 orders of magnitude lower than those required for STED microscopy using organic fluorophores. Despite obvious improvement in reducing pump intensities, nonetheless, previous experiments still required significant depletion beam intensities ranging from 100 kW*/*cm^2^ to tens of MW*/*cm^2^.^5,8,9,11^

The excitation intensities explored in this work do not exceed a few kW/cm^2^. Peak intensities at this level are sufficient to produce a noticeable narrowing of the spatial profile of the surplus emission compared to the profiles of either excitation beam (Figure S8). Notably, these intensities are 2 orders of magnitude lower than those employed in state-of-the-art STED experiments with UCNPs and over 4 orders of magnitude lower as compared to conventional STED microscopy.^13,14^ Therefore, despite complications related to the need for 2 images acquisition and simple image transformation (to be done *on the fly)*, the proposed method combined with the perfectly photostable and anti-Stokes UCNP luminescent labels is offering the potential to image light-sensitive samples over extended periods of time that would otherwise be damaged by the higher intensities required in conventional STED and UC-STED microscopies. As compared to the standard STED, the proposed UCNPs offer NIR excitation and NIR emission, which, although increasing the diffraction-limited beam size, offer unprecedented perspective for deep tissue imaging, with reduced light scattering, zero background signal, and simultaneous compatibility with other, fluorescence-based imaging modalities. As compared to conventional STED imaging, the expected optical resolution of the proposed method should be similar, while the acquisition times would be less competitive, mostly because of the requirement to acquire two images with combined beams (NIR with 975 and 975 nm).

When interpreting the above results, one has to take into account that the spectrum of the NIR beam is relatively broad (in terms of spectral width) compared to the absorption spectra of ions in UCNPs (especially at the long-wave end of the studied range; Figure S13). This spectral width is an intrinsic property of the tunable NIR light source used in the current work. Whereas it allowed the identification of coexcitation effects and suitable excitation wavelengths, only a fraction of the beam power is effectively exciting the relatively narrow-band excitation bands (c.a. 20–40 nm) within Tm^3+^ ions.

It should also be noted that the NIR beam used in our experiments is pulsed with a maximum repetition rate of 50 kHz. The observed effect depends on the pulse repetition period and increases rapidly as the pulse interval is reduced from 100 μs (corresponding to a repetition frequency of 10 kHz) to 20 μs (50 kHz). The shape of the surplus dependence on the pulse interval (Figure S9) strongly suggests that a much larger effect could be observed at higher pulse repetition rates or with continuous-wave (CW) lasers. A more detailed discussion and analysis of this aspect are provided in the Supporting Information.

Consequently, these two factors, namely, the spectral overlap between the lasers and the ESA bands, and the effect of the laser pulse repetition frequency, clearly indicate that the feasibility of the proposed applications could be significantly improved: the described coexcitation phenomena are expected to occur at substantially lower NIR intensities if a continuous-wave, narrow-band diode laser precisely matched to the relevant transitions is used.

When considering pulsed excitation and dynamic effects, it is worth noting that the experimental determination of luminescence kinetics under a coexcitation scheme could provide additional insights necessary for a complete understanding of the system. While such measurements are currently beyond our technical capabilities, we anticipate that coexcitation should lead to a reduction in luminescence rise times by opening additional channels for population transfer to higher excited states. Faster rise times could help overcome a major limitation of upconversion luminescent labels—their inherently slow response caused by the multistep excitation process, which restricts their broader application in raster-scanned imaging. A carefully timed sequence of 975 nm and NIR coexcitation pulses could potentially produce a faster emission rise than single-pulse excitation alone, although this remains to be experimentally confirmed.

Coexcitation also holds promise for alleviating a common limitation in lanthanide-doped upconverting nanoparticles (UCNPs), namely, concentration quenching, which arises from cross-relaxation (CR) processes between neighboring activator ions such as Tm^3+^. These nonradiative energy transfers become increasingly probable at higher dopant concentrations, leading to reduced emission efficiency. As a result, activator concentrations are typically kept low to minimize CR. However, the dual-beam coexcitation strategy may help overcome this constraint. Specifically, coexcitation can deplete the ground-state population of Tm^3+^ ions, thereby suppressing CR processes that require ground-state partners. In such a regime, higher dopant concentrations could become viable without a significant loss in emission efficiency.

Nevertheless, it should be noted that the energy migration and relaxation dynamics in lanthanide networks are highly complex and conditioned by past excitation parameters, involving collective interactions that go beyond pairwise CR mechanisms.^20^ Thus, experimental validation is necessary—such as systematic studies on UCNPs with varying Tm^3+^ contents, yet it can be expected that coexcitation may enable alternative design strategies for brighter and more robust UCNP-based systems with a reduced effect of concentration quenching.

Taken together, these opportunities highlight the versatility and potential of coexcitation to advance upconversion research and enable more efficient, adaptable systems.

## Conclusions

In this work, we investigated a previously underexplored yet highly promising strategy to modulate and enhance upconversion emission *in situ* via carefully selected coexcitation at two distinct near-infrared (NIR) wavelengths. Specifically, we observed that the simultaneous excitation of YbTm-codoped upconverting nanoparticles with a fixed 975 nm beam—resonant with Yb^3+^ absorption—and a broadly tunable NIR source spanning 1050–1875 nm, covering ground- and excited-state near-infrared absorption bands of Tm^3+^, leads to remarkable emission enhancement.

Through systematic spectroscopic analysis, we identified two key absorption bands of Tm^3+^ centered at 1213 and 1732 nm. Coexcitation at one of these wavelengths, in combination with a conventional 975 nm excitation, produced substantial emission enhancements of up to 600 and 800%, respectively. These enhancements far exceed the additive contributions expected from separate excitations and, in certain regimes, even result in visible upconverted emission, where neither excitation wavelength alone generates a detectable signal.

This striking phenomenon reveals the unique photophysical pathways accessible only through multiwavelength excitation and highlights coexcitation as a powerful tool for controlling and amplifying upconversion processes—opening avenues for applications in sensing, imaging, and NIR-to-visible photon conversion.

Taking advantage of the coexcitation-driven surplus emission phenomenon, we successfully demonstrated the direct visualization of infrared beams at wavelengths beyond the range of currently available technologies—in particular, surpassing the detection capabilities of standard InGaAs photodetectors beyond 1700 nm. Moreover, we have shown that these findings can support the development of new strategies for super-resolution microscopy. Specifically, our results suggest the feasibility of subdiffraction resolution imaging at beam intensities in the single kW/cm^2^ range—that is, well below the power densities required for existing direct super-resolution techniques. Although the surplus signal necessitates acquisition of two images, the proposed method offers unprecedented advantages: it should be possible to achieve subdiffraction resolution using sample irradiation at intensities 2 orders of magnitude lower than those employed in state-of-the-art STED microscopy with upconverting nanoparticles and 4 orders of magnitude lower than those required in conventional STED microscopy with organic labels. Furthermore, the anti-Stokes nature of the emission—where both excitation beams and the emitted light lie in the near-infrared range—ensures enhanced signal-to-background detection even in complex, highly scattering, and absorbing samples such as biological tissues. These features, combined with the perfect photostability of lanthanide-doped labels, offer the potential for extended observation times in fundamental biological studies.

Future research should focus on refining excitation wavelengths and optimizing nanoparticle design, including core–shell structures and dopant concentrations, to fully profit from this coexcitation strategy. In doing so, this approach paves the way for expanded opportunities in nanophotonics: enhancing the ability to convert invisible infrared signals into vibrant, multicolor light and enabling advanced techniques for long-term, background-free super-resolution imaging, highly localized photoactivation (e.g., photodynamic therapy, optogenetics), anticounterfeiting, and all-optical data processing.

## Methods

The hexagonal β-NaYF_4_ nanoparticles doped with 0.1% Tm^3+^ and 20% Yb^3+^ ions were prepared using a thermal decomposition reaction of lanthanide oleates.

### Preparation of Lanthanide Acetates [(CH_3_COO)_3_Tm, (CH_3_COO)_3_Yb (CH_3_COO)_3_Y]

Stoichiometric amounts of respective Y_2_O_3_, Tm_2_O_3_, and Yb_2_O_3_ lanthanide oxides (1.25 mmol) were mixed with a 50% aqueous acetic acid. The mixture was stirred and heated up to obtain a clear and transparent solution. The final precursor was obtained by evaporation of solvents at prevacuum and further drying at 140 °C for 12 h to obtain (CH_3_COO)_3_Ln powder.

### Preparation of the Nanoparticles

The acetate [(CH_3_COO)_3_Ln], 2.5 mmol, was added to the flask with 15 mL of oleic acid and 38 mL of octadecene. The solution was stirred and heated to 140 °C under vacuum for 30 min to form the oleate complex and to remove total oxygen and remaining water. Next, the temperature was lowered to 50 °C and 10 mmol of ammonium fluoride (NH_4_ F) and 6.25 mmol of sodium hydroxide (NaOH) were dissolved in 20 mL of methanol and added to the reaction flask. The resulting mixture was stirred for 30 min at 70 °C. Next, the reaction temperature was increased and the methanol was evaporated. After removing methanol, the solution was heated up to 300 °C under a nitrogen atmosphere and kept in such conditions for 1 h. Next, the mixture was cooled to room temperature. The nanoparticles were precipitated using ethanol, centrifuged at 10000 rpm for 10 min, and washed with hexane and ethanol. Finally, the prepared nanoparticles were dispersed in 5 mL of chloroform. A representative transmission electron microscopy (TEM) image and X-ray diffraction (XRD) pattern of the investigated nanoparticles are shown in Figure S10.

### Preparation of Samples

For spectroscopy measurements, the nanoparticles were washed with a 0.1 M hydrochloric acid solution to remove the surface organic layer, centrifuged at 16200 rpm for 10 min, redispersed in a small volume of water, and deposited onto microscope slides by drop-casting from the resulting suspension.

### Coexcitation of Nanoparticles

The experimental setup enabled the simultaneous excitation of samples using two spatially overlapped laser beams (Figure S11). The first beam, with a wavelength of 975 nm, was tuned to the ^2^F_7/2_ → ^2^F_5/2_ transition in Yb^3+^ ions and excited Tm^3+^ ions via an energy-transfer mechanism. The second beam, tunable in the range of 1050–1875 nm, was expected to interact directly with thulium ions, as no corresponding transitions exist for ytterbium ions within this spectral range.

For emission spectra and intensity measurements, samples were placed inside the Horiba QuantaMaster 8075–11 spectrofluorometer equipped with a PPD850 photomultiplier (sensitivity in the range 250–850 nm). The beam from a 975 nm diode laser (CNI, MDL-III-975–1000 mW) was combined with a beam tunable in the range of 1050–1875 nm, using a polarizing beamsplitter (Figure S11). Although both beams lie in the near-infrared region, in the article, only the latter is referred to as the *NIR beam*, owing to its tunability. A pair of lenses was placed in the path of the 975 nm beam to match the spatial profiles of both beams. The beams were aligned to be collinear by verifying their spatial overlap just after the beamsplitter and at the iris aperture positioned in front of the spectrofluorometer. Upon entering the sample chamber, the beams were focused onto the sample using a lens with a focal length of 50 mm. Dimensions of the beams at the focal point were determined directly inside the sample chamber using the knife-edge method. The following values of the beam waist at the 1/*e* ^2^ intensity level were obtained for the wavelengths relevant to the study (horizontal × vertical): at 975 nm, 25 × 30 μm; at 1213 nm, 18 × 33 μm; and at 1732 nm, 20 × 37 μm. For calculations of the mean excitation light intensity at the sample, the power of the beams was divided by the area of an ellipse with the vertical semiaxis equal to the vertical beam waist and the horizontal semiaxis equal to the horizontal beam waist multiplied by $\sqrt{2}$ . The latter factor accounted for the fact that the sample was placed at an angle of approximately 45° with respect to the beam directions. The final beam overlap was fine-tuned by monitoring the emission intensity while small adjustments were made to the position of one of the beams. The polarization of each beam was independently controlled using a half-wave plate, and variable neutral density filters enabled the adjustment of the power of each beam.

### Generation of Tunable Near-Infrared Beam

The NIR beam was generated in the process of difference frequency generation by mixing, in a BBO crystal, the output of an optical parametric amplifier (Orpheus by Light Conversion, output range 710–1010 nm) pumped by the output of a femtosecond amplifier (Carbide by Light Conversion, wavelength 1030 nm, maximum repetition frequency 50 kHz) with the second harmonic of the output beam of the same amplifier (Figure S12 in the Supporting Information). As a result, a train of femtosecond pulses with a central wavelength tunable in the range of 1050–1875 nm was generated. A long-pass filter was used to block the remaining light at shorter wavelengths. Unless otherwise noted, the maximum repetition rate of the system (50 kHz) was used. The mean output power of the NIR beam was wavelength-dependent, with a maximum of approximately 300 mW. When measurements were carried out for a range of NIR beam wavelengths, the excitation power at all wavelengths was equalized with the help of a variable neutral density filter (VNDF in Figure S11). Spectra of the NIR beam for selected wavelengths are shown in Figure S13 in the Supporting Information. The chosen method for generating the NIR beam, based on nonlinear frequency conversion of femtosecond pulses, enabled the continuous tuning of the excitation wavelength over a broad spectral range, which is not achievable with a single continuous-wave laser. This capability allowed the identification of transitions relevant to the coexcitation mechanism.

## Supplementary Material



## Notes

Emission spectra of nanoparticles; emission intensity as a function of excitation intensity—single excitation and coexcitation; spatial emission intensity analysis; analysis of potential for subdiffraction imaging; analysis of pulse repetition period; additional details of methods and schemes of the experimental setup (PDF)
