# Supplementary material for: Strong Emission Enhancement via Dual-Wavelength Coexcitation in YbTm-Doped Upconverting Nanoparticles for Near-Infrared and Subdiffraction Imaging
Source: ACS Nano. 2025 Jul 8;19(29):26932–41. doi: 10.1021/acsnano.5c08510 (PMC12312145; doi:10.1021/acsnano.5c08510)
Supplement: Supplementary file 1 [file nn5c08510_si_001.pdf]

Supporting Information: Strong Emission Enhancement via Dual-Wavelength Coexcitation in YbTm-Doped Upconverting Nanoparticles for Near-Infrared and Subdiffraction Imaging

Paulina Rajchel-Mieldzioć,† Artur Bednarkiewicz,‡ Katarzyna Prorok,‡ and Piotr Fita*,†

†*Institute of Experimental Physics, Faculty of Physics, University of Warsaw, Pasteura 5, Warsaw 02-093, Poland*

‡*Institute of Low Temperature and Structure Research, Polish Academy of Sciences, Okólna 2, Wrocław 50-422, Poland*

E-mail: fita@fuw.edu.pl

Emission spectra of nanoparticles

Figure S1 presents the emission spectra of the analyzed YbTm nanoparticles under single-beam excitation with a 975 nm laser (Figs. S1a and S1b), as well as under dual-beam coexcitation with either a 1732 nm (Figs. S1c and S1d) or 1213 nm (Figs. S1e and S1f) beam. For comparison, each coexcitation spectrum also includes the emission recorded under excitation by the 1732 nm and 1213 nm beams alone. However, the signal generated by these beams individually is negligible compared to excitation with the 975 nm beam or under coexcitation conditions; as a result, their corresponding traces lie close to the baseline.

As seen in the emission spectra under single-excitation with a 975 nm beam (Figure S1a), the $^3H_4 \longrightarrow {}^3H_6$ transition at 800 nm clearly dominates the emission profile. Due to the energy-level structure and intrinsic properties of Tm^{3+} ions in a $NaYF_4$ host lattice, this transition is inherently several times more intense than emissions originating from higher excited states. These higher-energy transitions—such as $^1G_4 \longrightarrow {}^3F_4$ (650 nm) and $^1G_4 \longrightarrow {}^3H_6$ (around 480 nm)—require additional population steps and are thus characterized by longer luminescence rise times. These are further convoluted with intrinsic lifetimes and can be significantly influenced by nonradiative depopulation (e.g., multiphonon relaxation), surface quenching (via ligand or solvent vibrations), or concentration quenching due to ion-ion interactions.

Moreover, under coexcitation, the 800 nm emission band also exhibits the largest relative enhancement—further solidifying its position as the dominant feature in both absolute and relative terms. For the sake of clarity and interpretive consistency, we chose to focus our analysis on this most intense transition ($^3H_4 \longrightarrow {}^3H_6$), whose enhancement under coexcitation was likewise the most pronounced.

That said, other emission bands also experience enhancement under dual-beam excitation. The ultraviolet transitions ($^1I_6 \longrightarrow {}^3F_4$ and $^1D_2 \longrightarrow {}^3H_6$) are amplified—particularly under coexcitation with the 1732 nm beam. Under 1213 nm coexcitation, the effect is less prominent, especially for the $^1I_6 \longrightarrow {}^3F_4$ transition, which shows little enhancement under

the given conditions. However, due to the initially low emission intensities under 975 nm single excitation at the chosen intensity, even after enhancement, the absolute UV signal remains weak.

The blue emission bands ($^1D_2 \longrightarrow {}^3F_4$ and $^1G_4 \longrightarrow {}^3H_6$) are likewise enhanced—particularly the latter, which is more dominant and shows a stronger response under co-excitation with the 1732 nm beam. Nonetheless, its enhancement remains notably smaller than that of the 800 nm band, reaching at most about half the relative increase.

A similar trend is observed for the red emission bands ($^1G_4 \longrightarrow {}^3F_4$ and $^3F_3 \longrightarrow {}^3H_6$), both of which are enhanced under coexcitation with either NIR wavelength. However, the $^3F_3 \longrightarrow {}^3H_6$ transition in particular shows a stronger response to coexcitation with the 1732 nm beam.

All these trends point to a broader observation: while all emission bands are enhanced under coexcitation, the process disproportionately amplifies the already dominating 800 nm emission band (compare Figure S1a with Figures S1c and S1e). This is the reason we selected that transition to serve as the central focus of our study. Nonetheless, it remains important to acknowledge that the other emission bands also exhibit clear—and often substantial—enhancement, presenting additional avenues for mechanistic exploration, ideally guided by the insights gained from our detailed study of 800 nm photophysics in the coexcitation regime.

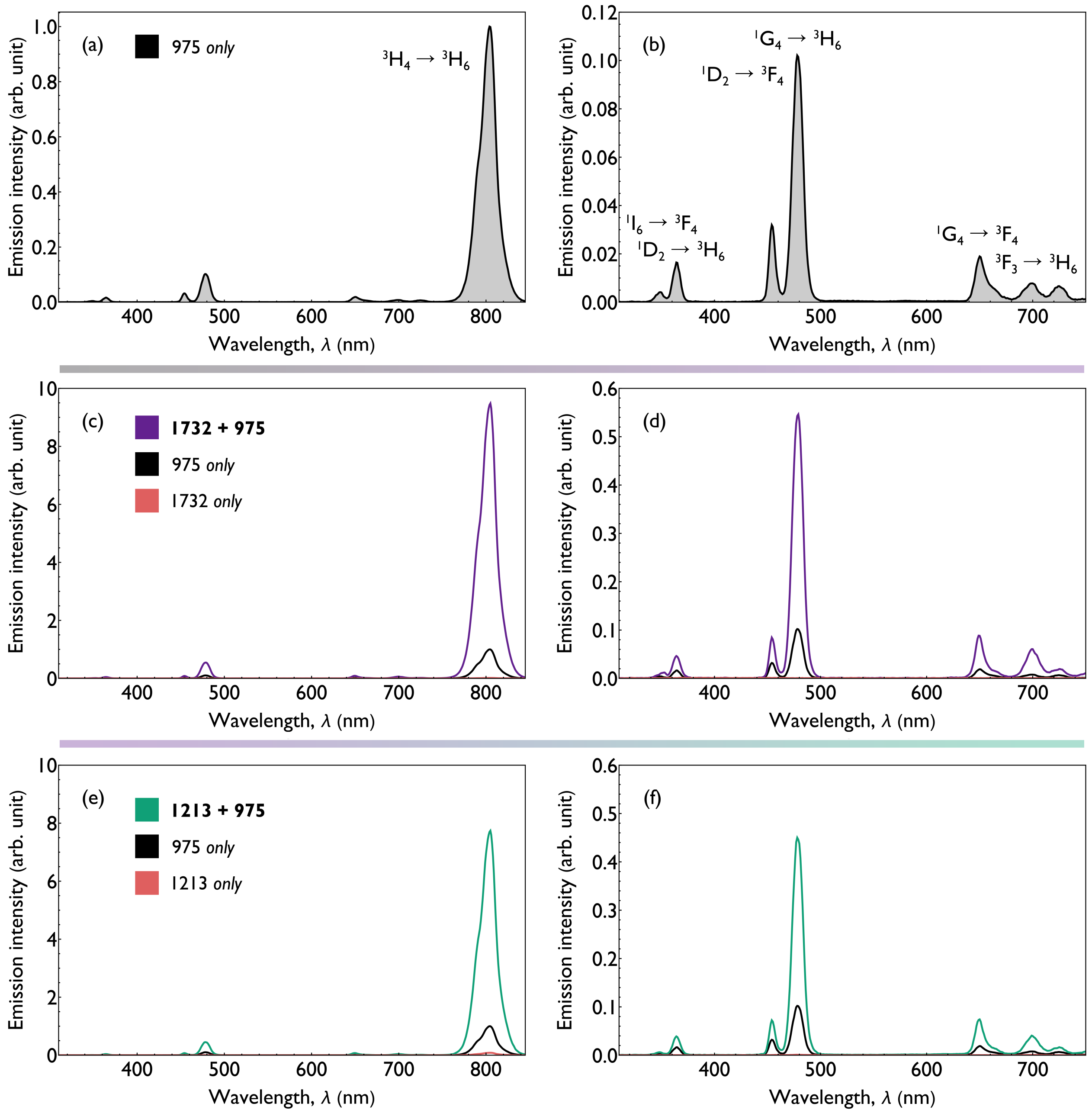

Figure S1: Emission spectra of the analyzed YbTm nanoparticles. **(a)** Emission spectrum under 0.3 kW/cm^2 (10 mW) continuous-wave 975 nm excitation, showing a clearly dominant emission band around 800 nm. **(b)** Magnified view of the emission spectrum from (a), limited to the range below 750 nm. **(c)** Emission spectrum under coexcitation with 0.3 kW/cm^2 (10 mW) continuous-wave 975 nm and 3.0 kW/cm^2 (100 mW) 1732 nm beams. For comparison, emission induced by the 975 nm beam alone (black line) and the 1732 nm beam alone (coral line, near the baseline) is also shown. **(d)** Magnified view of the emission spectrum from (c), limited to the range below 750 nm. **(e)** Emission spectrum under coexcitation with 0.3 kW/cm^2 (10 mW) continuous-wave 975 nm and 3.8 kW/cm^2 (100 mW) 1213 nm beams. For comparison, emission from the 975 nm beam alone (black line) and the 1213 nm beam alone (coral line, near the baseline) is included. Magnified view of the emission spectrum from (e), limited to the range below 750 nm. All spectra are shown on the same scale to facilitate comparison.

Emission intensity as a function of excitation intensity

Single-source excitation

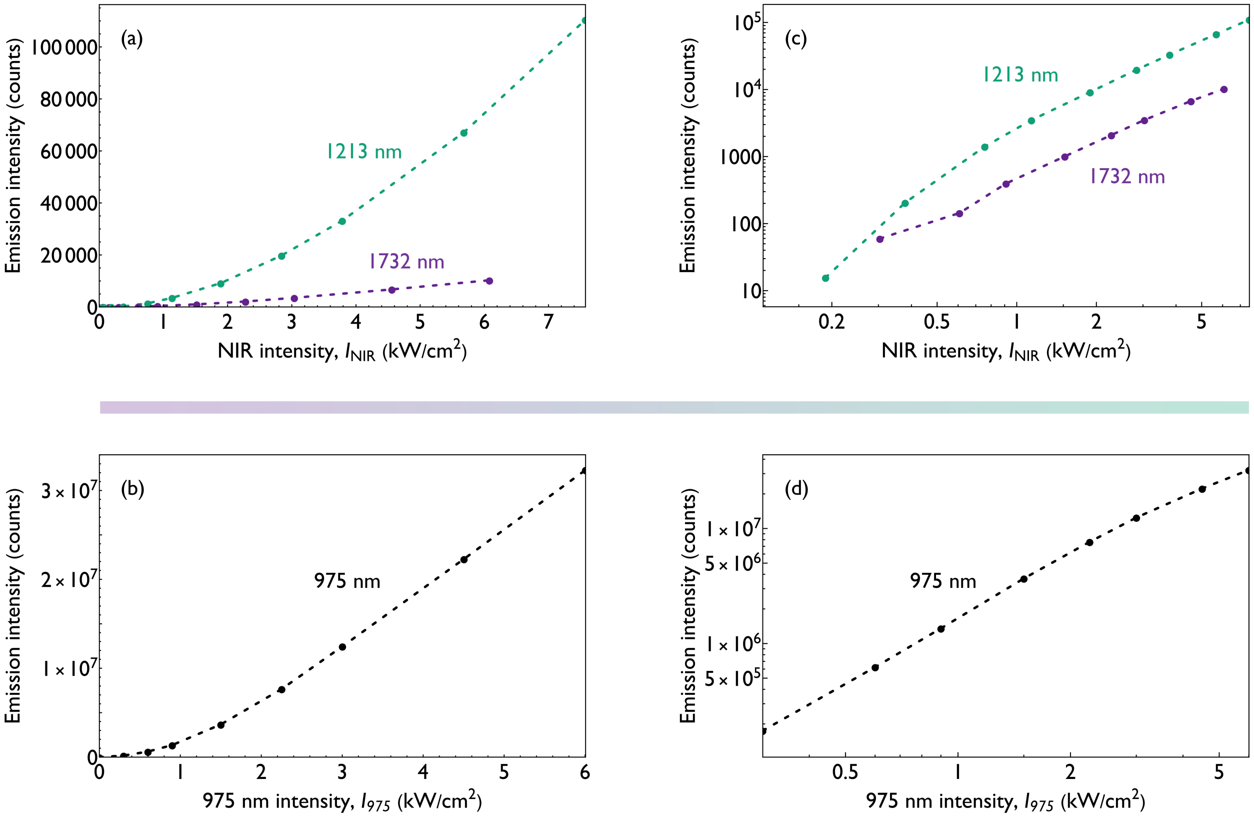

Figure S2: Emission intensity recorded at 800 nm from YbTm nanoparticles under single-source excitation, plotted as a function of excitation intensity—shown on both a linear scale (left) and a log-log scale (right). Data points corresponding to zero emission counts are omitted from the log-log plots, as they cannot be represented on a logarithmic scale. **(a)** Emission under excitation with NIR beam (violet: 1732 nm, green: 1213 nm), plotted as a function of NIR beam intensity. **(b)** Emission under excitation with a 975 nm continuous-wave beam, plotted as a function of beam intensity. Note the difference in vertical scale between the two graphs. **(c)** Same as (a), plotted on a log-log scale. **(d)** Same as (b), plotted on a log-log scale. Dashed lines connecting data points are included for visual clarity.

Coexcitation

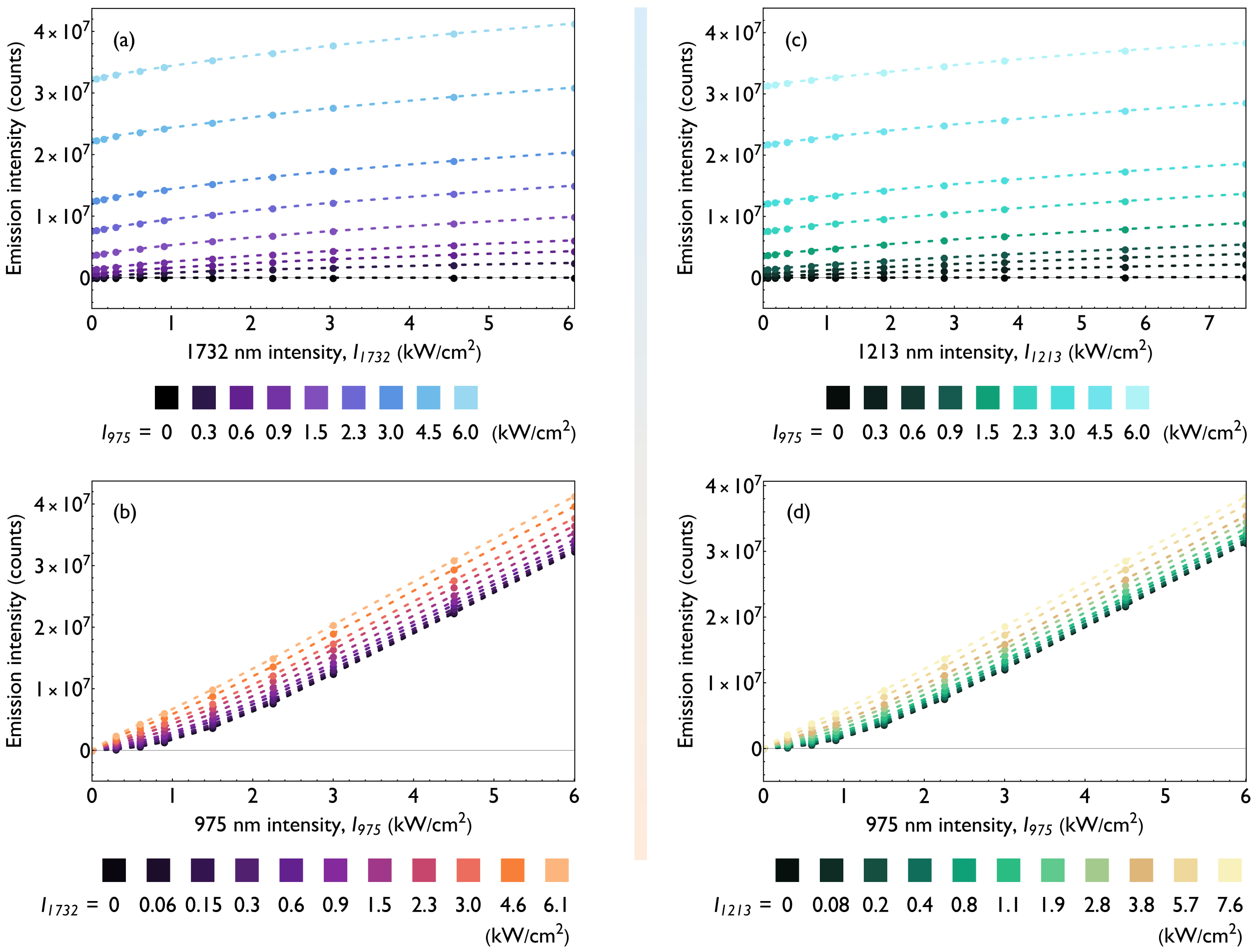

Figure S3: Emission intensity recorded at 800 nm from YbTm nanoparticles under coexcitation with 975 nm and NIR beams, shown as a function of excitation intensities. **(a)** Emission under simultaneous excitation with 975 nm and 1732 nm beams, plotted as a function of 1732 nm beam intensity for various levels of 975 nm intensity. **(b)** Complementary plot: emission as a function of 975 nm beam intensity, for various levels of 1732 nm intensity. **(c)** Emission under simultaneous excitation with 975 nm and 1213 nm beams, plotted as a function of 1213 nm beam intensity for various levels of 975 nm intensity. **(d)** Complementary plot: emission as a function of 975 nm beam intensity, for various levels of 1213 nm intensity. Dashed lines connecting data points are added for visual clarity. Axes have been adjusted to improve visibility of near-zero emission values.

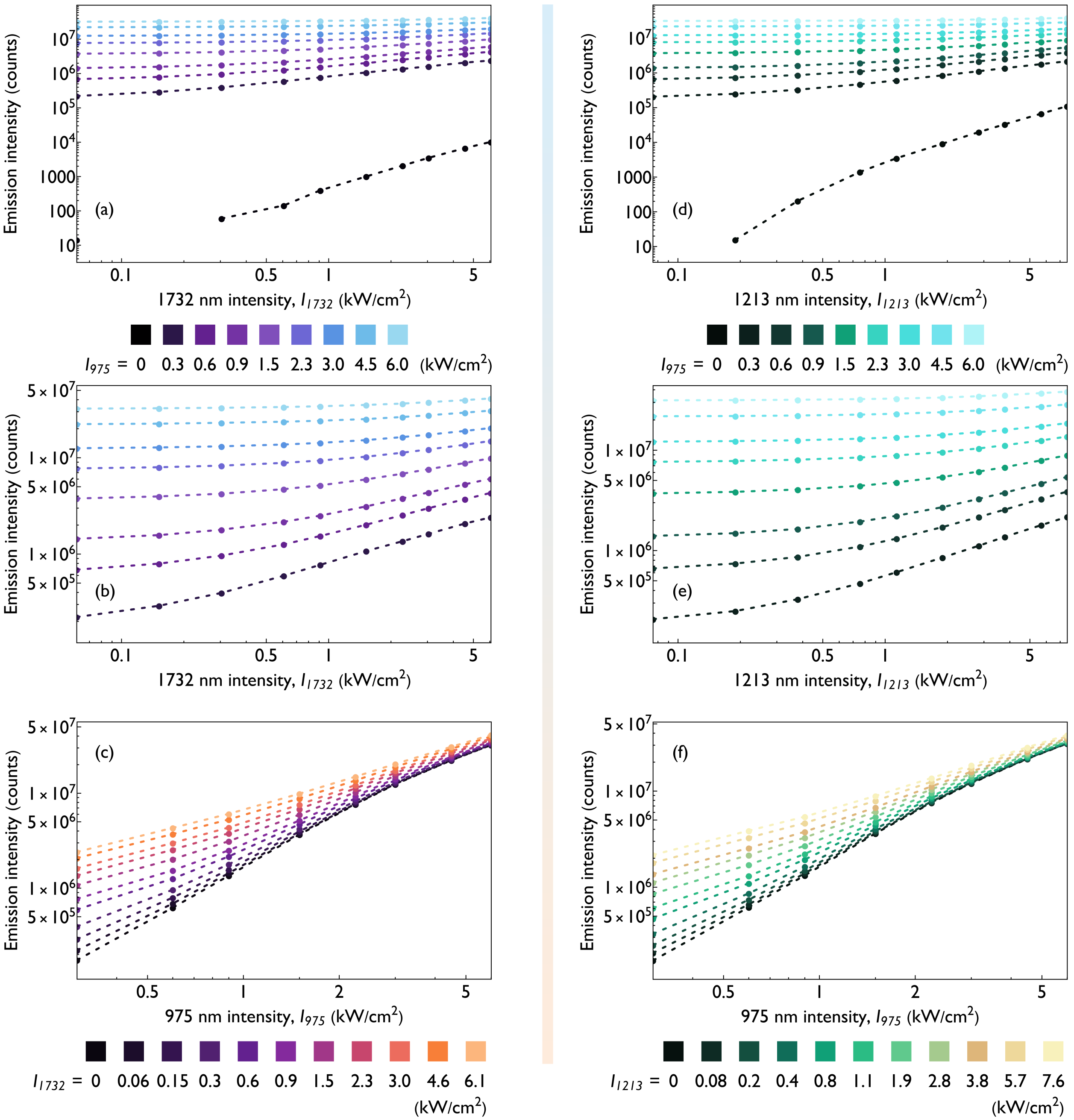

Figure S4: Same data as in Figure S3, but presented on a log-log scale. Emission intensity at 800 nm from YbTm nanoparticles under coexcitation with 975 nm and NIR beams, shown as a function of excitation intensity. Note that data points corresponding to zero intensity on the x-axis or zero emission counts are excluded, as they cannot be displayed on a logarithmic scale. **(a)** Emission intensity under simultaneous excitation with 975 nm and 1732 nm beams, plotted as a function of 1732 nm intensity at various 975 nm intensity levels. **(b)** Same as (a), but with the 0 kW/cm^2 975 nm series omitted for clarity. **(c)** Complementary plot: emission intensity as a function of 975 nm intensity at various 1732 nm intensity levels. **(d)** Emission intensity under simultaneous excitation with 975 nm and 1213 nm beams, plotted as a function of 1213 nm intensity at various 975 nm intensity. **(e)** Same as (d), with the 0 kW/cm^2 975 nm series omitted for clarity. **(f)** Complementary plot: emission as a function of 975 nm intensity at various levels of 1213 nm intensity. Dashed lines connecting data points are added for visual clarity.

Coexcitation in the low-intensity regime

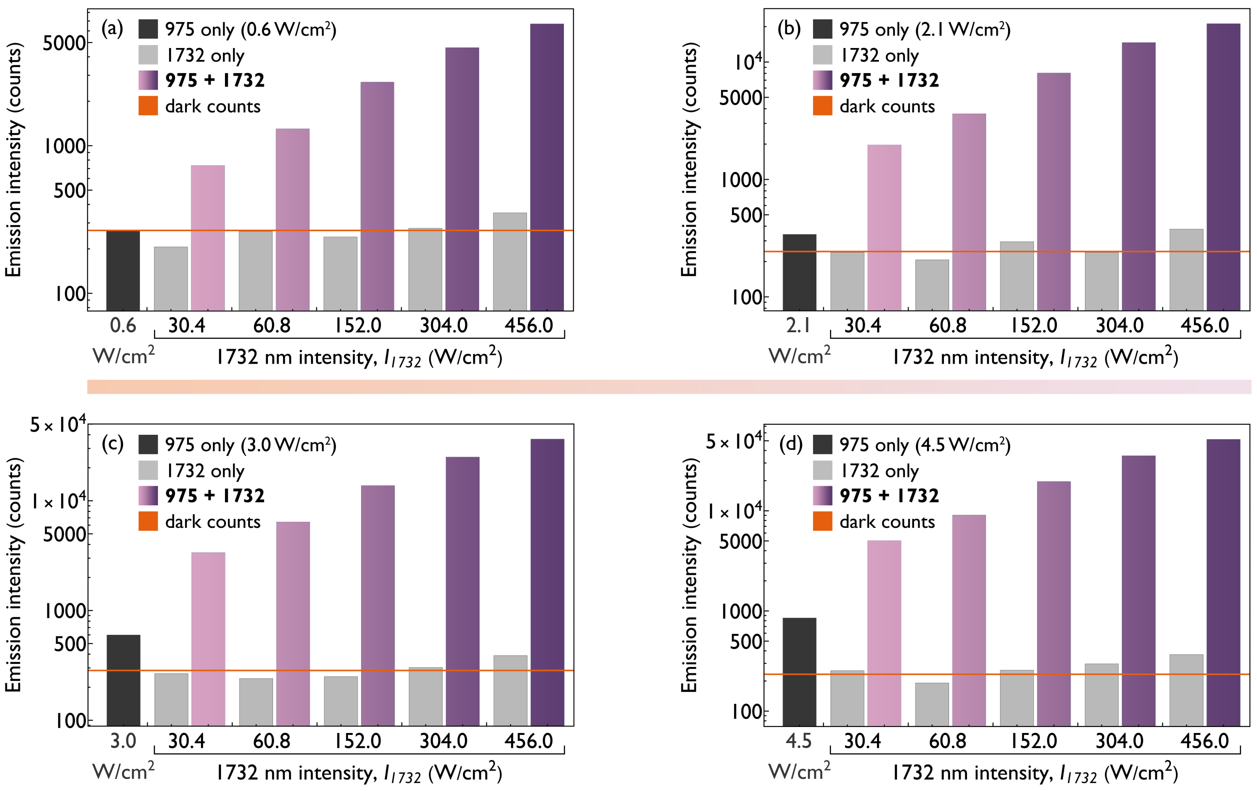

Figure S5: Analysis of emission intensity recorded at 800 nm under coexcitation with weak 975 nm and 1732 nm beams (purple bars), collected for a range of 1732 nm beam intensities —**presented on a log scale**. Dark gray—emission counts under excitation with only the 975 nm beam, light gray— emission counts under excitation with only the 1732 nm beam. Orange line—dark counts. **(a-b)** The same data, as in Figure 4a,b in the main text, shown on a log scale—with a 975 nm beam intensity of (a) 0.6 and (b) 2.1 W/cm^2, while varying the intensity of the 1732 nm beam. **(c-d)** Experiment performed with a 975 nm beam intensity of (c) 3.0 and (d) 4.5 W/cm^2, while varying the intensity of the 1732 nm beam, results shown on a log scale.

Spatial emission intensity analysis

Spatial emission intensity analysis was performed on the original, unscaled image to avoid artifacts caused by image rescaling. Brightness was defined as the arithmetic mean of the R, G, and B values of each pixel, as shown in eq S1:

$$\text{Brightness} = \frac{R+G+B}{3} \tag{1}$$

The maximum possible (arbitrary) brightness value is 255. In our case, no pixel reached this value, indicating that saturation did not occur. Figure S6 also shows an additional cross-section along the axis perpendicular to the one presented in the main text. The Gaussian fit in this case is also of good quality, and both fits yield very similar FWHM values.

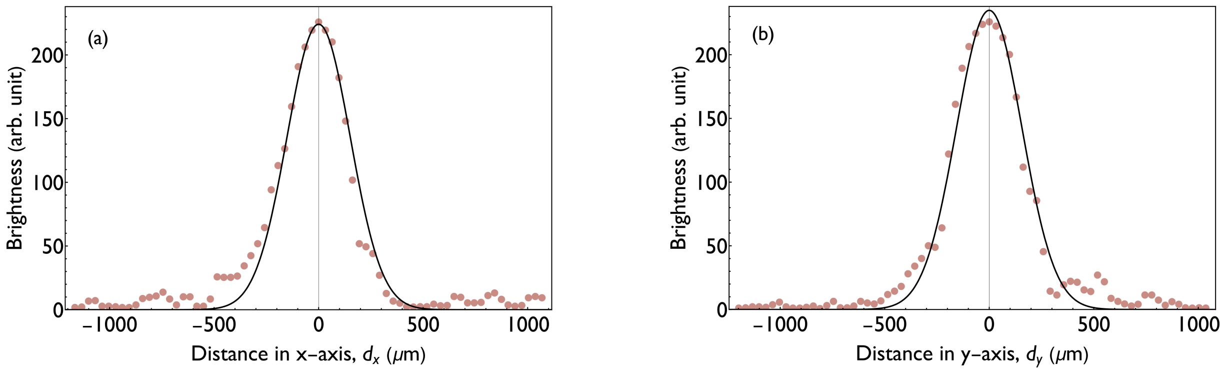

Figure S6: Emission intensity analysis based on brightness from the direct visualization of the 1732 nm beam using YbTm nanoparticles under weak 975 nm illumination (Figure 5 in the main text), with an additional cross-section. **(a)** A cross-section along the x-axis was taken through the intensity maximum in the visualization image (defining the zero point) and plotted against the actual spatial distance. A Gaussian fit is included (FWHM = **355**(13) μm). This graph also appears in the main text (Figure 5). **(b)** A cross-section along the y-axis, also through the intensity maximum, is plotted against the actual spatial distance, with a Gaussian fit FWHM = **369**(11) μm.

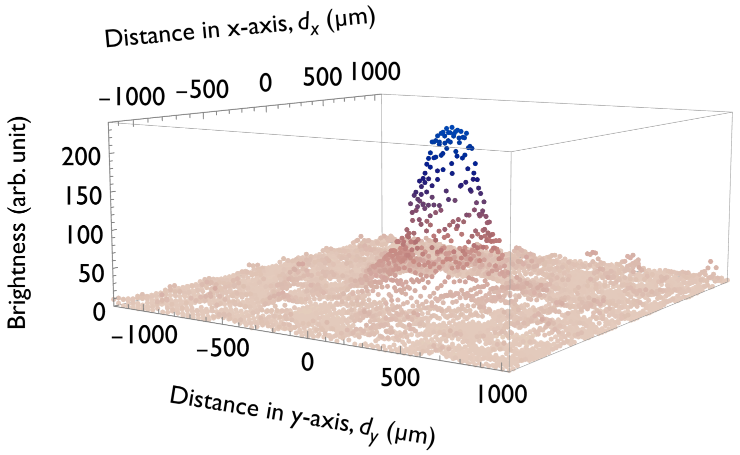

Figure S7: Two-dimensional representation of emission intensity (pixel brightness) from the the direct visualization of the 1732 nm beam using YbTm nanoparticles under weak 975 nm illumination (entire top central image in Figure 5 in the main text).

SNR analysis

The signal-to-noise ratio (SNR) was calculated as the ratio of the signal maximum (in brightness scale) to the noise, where the noise was estimated as the standard deviation of the first and last 10 pixels in the intensity profile. The analysis was performed for both cross-sections: the x-axis profile yielded an SNR of 64 while the y-axis profile resulted in an SNR of 71. Both values are high and comparable, confirming that the signal in the presented visualization (Figure 5 in the main text) is clearly and reliably distinguishable.

Potential for subdiffraction imaging

As indicated by eq 2 in the main text, the surplus emission is proportional to the intensity of the NIR beam and inversely proportional to that of the 975 nm beam. When the spatial profiles of the two beams are appropriately tailored—for example, using a Gaussian profile for the NIR beam and a donut-shaped profile for the 975 nm beam—the resulting spatial distribution of the surplus emission can be significantly narrower than either of the individual beams, as illustrated in Figure S8. This effect offers potential for imaging with subdiffraction

resolution.

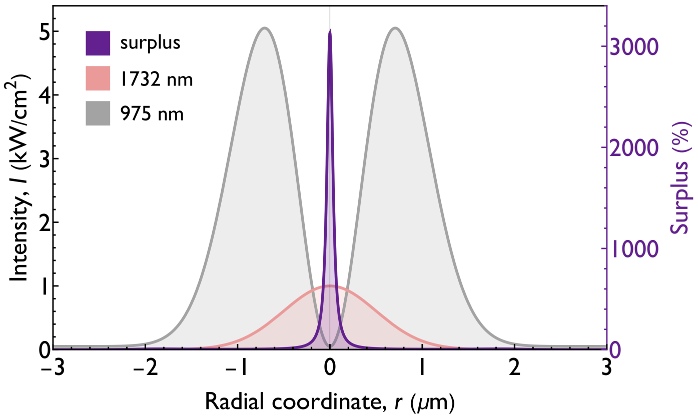

Figure S8: Plot of the radial distribution of the surplus emission $S(r)$, eq 5 (violet), along with radial profiles of the 1732 nm ($I_{\mathrm{NIR}}(r)$, Gaussian – pink), and 975 nm ($I_{975}(r)$, Laguerre-Gaussian with $l = 1$, $p = 0$ – grey) beams. The waist radius of both 1732 nm and 975 nm beams is equal to 1 μm, $x = 0.69$ and $y = -1.20$ according to eq 3, and the intensity in the center of the 975 nm beam is equal to 0.01 of the maximum intensity.

Influence of pulse repetition period

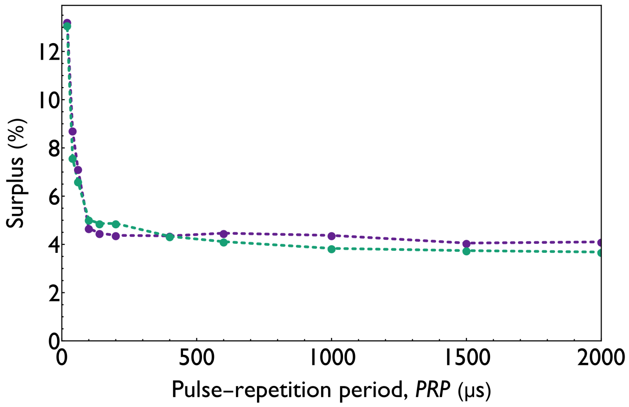

Figure S9: Surplus 800 nm emission obtained under coexcitation of the sample with a 975 nm beam (8.2 mW, 0.25 kW/cm^2) and a pulsed NIR beam (1 mW, 0.03 kW/cm^2 for 1732 nm, violet and 1.1 mW, 0.04 kW/cm^2 for 1213 nm, green) as a function of the NIR pulse repetition period. The dashed lines connecting individual points have been added for clarity.

Since the experiments demonstrated in the main text employed a pulsed NIR source, the influence of the NIR beam's repetition rate was also investigated while maintaining a con-

stant average intensity. To adequately cover a broad range of repetition rates—including low-frequency values—a relatively low average intensity of the pulsed beam was required. Therefore, measurements were conducted with a continuous-wave 975 nm beam (8.2 mW, 0.25 kW/cm^2) combined with a pulsed NIR beams at reduced average power (1 mW, 0.03 kW/cm^2 for 1732 nm and 1.1 mW, 0.04 kW/cm^2 for 1213 nm). The dependence of surplus emission on the pulse repetition period (the inverse of repetition frequency, chosen for clarity) is presented in Figure S9. In particular, at short pulse intervals (high repetition rates), even at such low NIR beam intensity, the observed emission surplus was significant, exceeding 13%. As the interval between pulses increased, the effect systematically declined—a trend observed for both NIR wavelengths, 1732 nm and 1213 nm, equally. This behavior aligns with the upconversion mechanism, where the long-lived $^3F_4$ level plays a key role. This level is directly pumped by the 1732 nm beam and indirectly populated by the 1213 nm beam. When the interval between pulses exceeded 100 μs, the effect stabilized at a low level, and the NIR beam ceased to play a significant role in the upconversion ladder, which became dominated by the continuous 975 nm beam.

The rapid increase in surplus emission observed with decreasing pulse repetition periods indicates that even stronger effects might be achieved using a continuous-wave NIR beam. In particular, using a narrow-band continuous-wave NIR laser, with wavelength selection guided by the results presented here, should allow these phenomena to occur at beam intensities at least an order of magnitude lower than those required by the broadband pulsed NIR source utilized in this study.

Methods

Structural characterization of nanoparticles

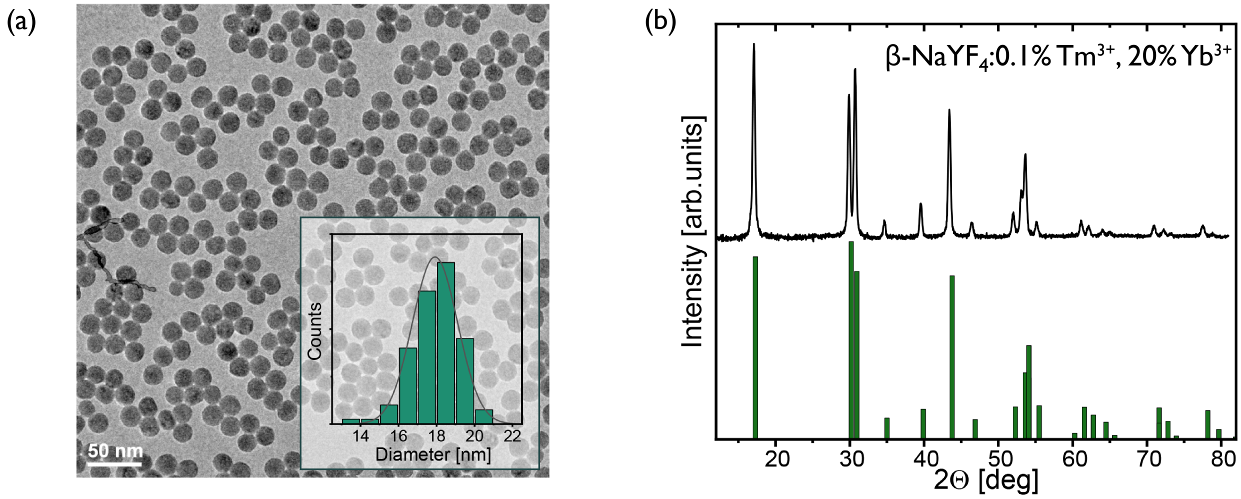

Figure S10: **(a)** Transmission electron microscopy (TEM) image of the investigated nanoparticles, showing their morphology and size distribution. Scale bar included. **(b)** X-ray diffraction (XRD) pattern of the investigated nanoparticles.

Experimental setup

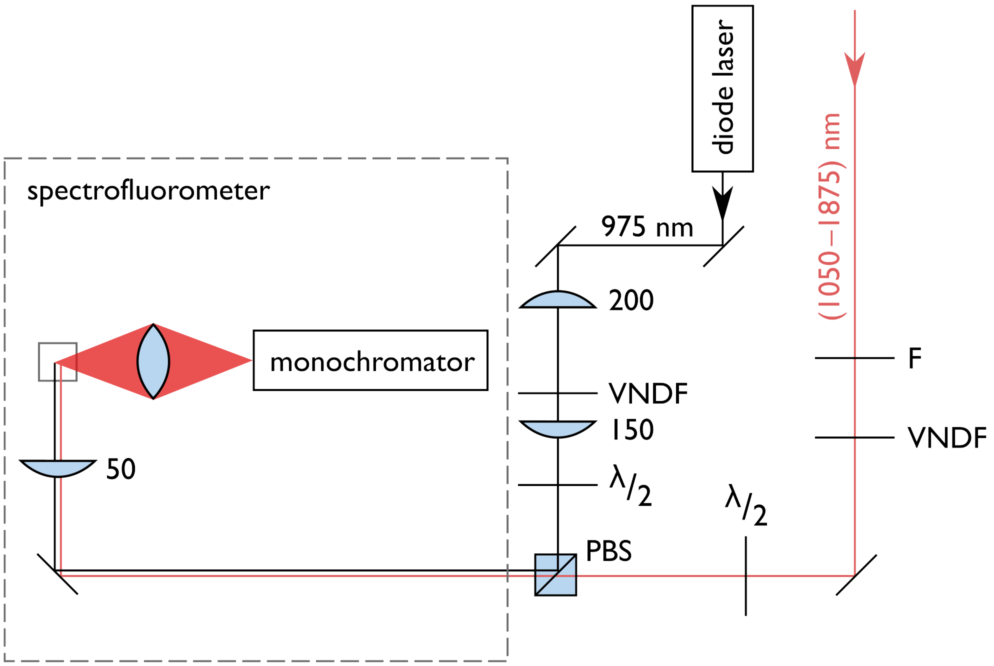

Figure S11: Schematic of the experimental setup for coexcitation measurements. The NIR beam (1050-1875 nm), generated by difference-frequency generation (DFG), is spatially overlapped with a continuous-wave 975 nm beam at a polarizing beam splitter. The combined beams are then directed into the spectrofluorimeter chamber, where the sample is excited and the resulting emission (800 nm) is recorded. Each beam can be switched off independently, allowing measurements with either single- or dual-beam excitation.

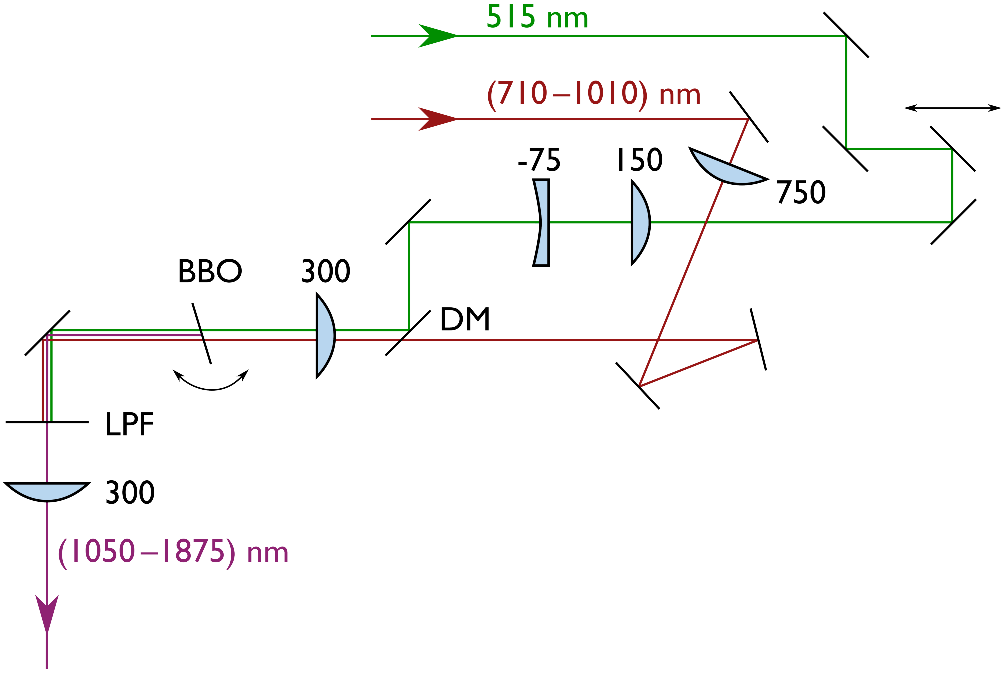

Figure S12: Schematic of the difference-frequency generation (DFG) setup used to produce a NIR beam (1050-1875 nm). Two pulsed beams—a 515 nm pump and a tunable signal output of an optical parametric amplifier (710-1010 nm)—are temporally overlapped using a delay line, then overlapped spatially on a dichroic mirror (DM) and finally focused in a β-barium borate (BBO) crystal. After precise phase matching in the BBO crystal, difference-frequency generation occurs. The generated NIR beam passes through a long-pass filter (LPF) to remove residual pump and signal wavelengths, and is subsequently directed to the spectrofluorometric setup.

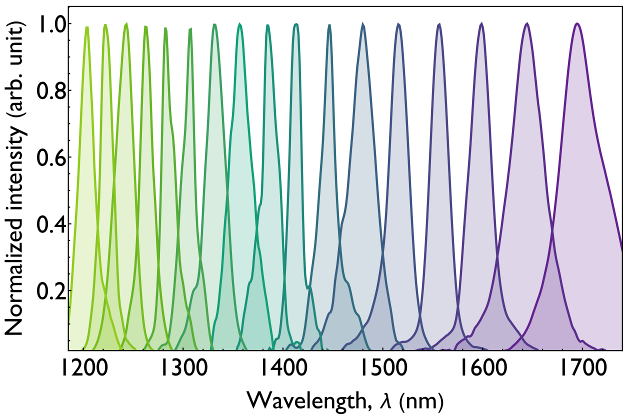

Figure S13: Normalized spectra of NIR pulses generated in the DFG system.
